# The Meaning of Work and Self-Management Experiences among Elderly Workers with Multiple Chronic Diseases: A Qualitative Study

**DOI:** 10.3390/healthcare8040471

**Published:** 2020-11-09

**Authors:** Juah Kim, Jiyeon Ha

**Affiliations:** 1 Department of Nursing, Korean Bible University, Seoul 01757, Korea; jooa04@bible.ac.kr; 2 College of Nursing, Konyang University, Daejeon 35365, Korea

**Keywords:** self-management, self care, multiple chronic conditions, chronic disease, aged, occupational groups, work, qualitative research

## Abstract

With population aging, increasingly many elderly individuals are expected to participate in economic activities. Elderly workers have a higher prevalence of multiple chronic diseases, making it necessary to examine elderly workers’ experiences of health-related self-management in work environments. This qualitative study investigated the meaning of work and health-related self-management experiences among elderly workers with multiple chronic diseases. The study participants were elderly workers residing in South Korea (65 years old or older) with at least two chronic diseases. Twelve participated in individual interviews, which were audio-recorded and transcribed. Qualitative content analysis was conducted with the transcribed data. Six themes, 21 sub-themes, and 40 codes were derived. The themes were “benefit of work on health and life”, “adaptation to a new work environment”, “endurance”, “continuous efforts to maintain health”, “difficulties in self-management”, and “requirements for health promotion”. The participants experienced difficulties in health-related self-management due to time constraints, poor work environment, and financial burdens. However, participants expressed their desire to invest effort into managing their physical and psychological health and to work as long as possible. Programs that consider the aging and health-related characteristics of elderly workers and their work environments should be developed and implemented.

## 1. Introduction

As the elderly population grew due to improvements in medical technology and longer lifespans, the working life expectancy increased worldwide, leading to a greater number of elderly workers [1,2]. Old age used to be perceived as a time to retire and look back on the life that one has lived, but this paradigm is shifting as the population continues to age. In the United States, the number of elderly workers has been increasing dramatically since 2000 [3], and in Europe, the inflow of young workers has slowed down while the retirement age has gradually gone up [4]. South Korea is experiencing various social issues due to rapid population aging, including poverty among the elderly due to insufficient planning for retirement [5]. The current generation of elderly individuals in South Korea faced difficulties in saving for retirement due to the need to support their parents as well as their children and their education. An earlier retirement age and the lack of a social safety net have made it necessary for many elderly individuals to continue working to survive and meet their economic needs [5]. According to a 2017 Ministry of Health and Welfare report, 30.9% of individuals aged over 65 were currently working, and 73.0% of those who were working indicated that the reason for working was to cover living expenses [6]. Other reasons included compensating for losses in their retirement savings accounts [7]. Nonetheless, this is a global issue, as the proportion of elderly participating in economic activities is expected to continue to increase concomitantly with population aging throughout the world [4,7]. 

It has been reported that about 73% of individuals over the age of 65 have multiple chronic diseases, and have an average of 2.7 chronic diseases [6]. More than half of elderly workers have multiple chronic diseases [6], and may experience difficulties in health-related self-management due to the impact of those diseases on their physical capacity [8]. A systematic review on work-related illness and accidents among individuals aged 60 or above found that the risk of accidents increased due to mobility and sensory (auditory and visual) loss in the elderly [9]. In the South Korean labor market, elderly workers are generally low-wage workers in occupations such as sanitation, security, and construction [6]. Elderly workers’ vulnerabilities in terms of their physical condition and work environment are therefore important occupational health issues [4,10]. 

To date, research on self-management among elderly workers (above the age of 65) in South Korea who have multiple chronic diseases, for whom self-management of health is critical, is still lacking aspects of the work environment that could cause the health condition of elderly workers with chronic diseases to deteriorate, and various work characteristics can interfere with the self-management of chronic diseases [11]. Therefore, it is necessary to conduct a detailed analysis of the meaning of work and health-related self-management experiences of elderly workers who need continuous self-management of multiple chronic diseases. The population of elderly workers has increased, and this study provides insights into the meaning of work and experiences of health-related self-management among elderly workers who have multiple chronic diseases. The study will influence the development of health promotion programs that take into consideration the characteristics of elderly workers and the implementation of work environments that are health-friendly for the elderly. The aim of this study is to explore the meaning of work and health-related self-management experiences of elderly workers with multiple chronic diseases, thereby yielding insights into the meaning of work and health and requirements for self-management of health.

## 2. Materials and Methods 

### 2.1. Study Design

This qualitative study aimed to contribute to the health promotion of elderly workers by describing the meaning of work and health among elderly workers with multiple chronic diseases, understanding their health-related self-management experiences in depth. 

### 2.2. Participants

The participants in this study were elderly workers (aged 65 or above) who had two or more chronic diseases, defined as diseases lasting for at least 3 months. Participants were recruited in Seoul and Daejeon (urban areas) and required to understand the aim of the research and give voluntary consent. In order to improve the validity of the study, elderly workers who had been at their current positions for less than 3 months or who had been diagnosed with a cognitive disability were excluded. Purposive sampling was conducted to include participants with a wealth of information who could help identify the meaning of work and health-related self-management among elderly workers in an insightful manner [12]. Sampling was conducted until enough information was gathered to replicate the research and further coding was no longer feasible based on an analysis of participants’ interviews [13]. Twelve participants were included in this study. 

Seven of the study participants were women and five were men. Their age ranged from 65 to 74. Half of the participants (*n* = 6) had an elementary education, and the average monthly income was from 843 to 1687 USD. Participants engaged in various occupations, and included sanitation workers, security guards, office workers, and certified nursing assistants. Eight participants were Christians, two were Catholic, and the rest were Buddhists. Five participants lived with their children, and three lived alone. The number of comorbid chronic diseases ranged from 2 to 4, with an average of 2.83. The reported chronic diseases included hypertension, diabetes, hyperlipidemia, degenerative arthritis, osteoporosis, asthma, tinnitus, and depression (Table 1). 

### 2.3. Interviews and Procedures

#### 2.3.1. Research Team

The research team was composed of researchers actively conducting research on elderly workers who, as members of the Academy of Qualitative Research in South Korea, had participated in six seminars on qualitative research. The researchers had also gained credits for qualitative research methods in graduate school, and obtained knowledge about research planning and data analysis by reading the literature on qualitative research methods. All members also had experience participating in interviews and writing reports under the supervision of a senior qualitative researcher. The corresponding author participated as a co-author of another qualitative study in 2015 and published a qualitative research paper on the subject of self-management of nursing home residents. Furthermore, the research members discussed interview techniques for data collection and data analysis in regular meetings. 

#### 2.3.2. Interviews 

Data collection took place from 7 January to 12 February 2020 in Seoul and Daejeon, South Korea. A structured survey gathered information on the general and health-related characteristics of the participants. Individual in-depth interviews were selected as the main data collection method, and facial expressions, attitude, gaze, and intonations during the interview were observed and recorded in field notes. Quiet locations such as meeting rooms and seminar rooms that allowed for a focused discussion in a comfortable environment were selected prior to the interviews. Chairs were arranged so that the researcher and the participant were facing each other, and the participant was notified that audio-recording would take place once more before the researcher started to record. In order to prevent losing data, 2 recorders were used, and the recording was checked right after the interview. Each participant participated in one interview, and if additional information was required, the researchers called the participant on the phone. The duration of the interviews ranged from 50 to 90 min. Since the participants were elderly, close attention was paid to their physical condition, including whether they experienced dizziness and low energy levels. The interviews started with unstructured and open-ended questions such as “What is the meaning of work for elderly workers?” and “What is the meaning of health for elderly workers?” and moved on to more specific questions about health-related self-management experiences and requirements for health promotion (Table A1). 

#### 2.3.3. Transcripts

All interviews were audio-recorded with the agreement of the participants, and the audio files were saved with the participants’ ID. The files were listened to repeatedly and transcribed. After transcription was complete, the transcript was compared to the audio file to ensure accuracy of the transcription. Transcriptions were printed out, read carefully, and coded for analysis. 

#### 2.3.4. Ethical Aspects

This study was approved by Konyang University Institutional Review Board (IRB No. KYU-2019-277-01) before data collection. The participants were limited to those who voluntarily expressed intent to participate after researchers explained the aim and content of the study, and it was explained that discontinuing participation would not lead to any disadvantage. Written consent forms that contained the above information were provided to the participants. Participants were informed beforehand that the interviews would be audio-recorded, and precautions were taken to protect anonymity. After the interview, a token of appreciation was provided to participants. Only the research team could access the collected data, and field notes and transcripts were stored in a secure location with double locks inside the office of the research team. According to the regulations of the Institutional Review Board of Konyang University, the research materials will be destroyed 3 years after the conclusion of the research, and the collected data will only be used for research purposes. 

#### 2.3.5. Analysis

The recorded interviews were transcribed, and the text data were analyzed through Sendeloski’s qualitative content analysis. Qualitative content analysis is widely used when the aim is to interpret meaning from the text and explain the research phenomena in a straightforward manner [14]. Qualitative content analysis has three approaches, which are conventional, directed, and summative content analysis [15]. This study used conventional content analysis, where results are derived from the text of study participants directly [15]. First, researchers familiarized themselves with the data by reading the transcripts and field notes repeatedly. Second, meaningful and important parts of the data were extracted as codes. Third, codes were grouped together and organized. Fourth, the classified codes were condensed into themes or categories. In order to check whether the results of the analysis reflected participants’ experiences, the researchers shared feedback and opinions throughout the process to enhance the validity of the study results. 

#### 2.3.6. Rigor

The rigor of this study was evaluated following Lincoln and Guba’s model of trustworthiness, which includes truth value, applicability, consistency, and neutrality [16]. For truth value, member checking was conducted by randomly selecting a participant to hear the results and checking whether the results matched the participant’s experience. For applicability, which is equivalent to external validity in quantitative studies, data collection and analysis continued until saturation was reached, and all aspects of elderly workers’ meaning of work and experiences with self-management were captured as multiple dimensions and themes. For consistency, which is equivalent to reliability, the process of data analysis and interview questions were described in detail; voice recordings, transcripts, and field notes were used; and transcriptions were done as soon as possible after the interviews in order to ensure data would not be missing or distorted. In order to obtain neutrality, which is equivalent to objectivity, researchers must refrain from making judgments so that their prejudices are not involved in the process of collecting and analyzing raw data. These efforts were made for the results to reflect the essential components of work and vivid self-management experiences as closely as possible. 

## 3. Results

The data collected from 12 participants were analyzed according to Sendeloski’s qualitative content analysis method, and as a result, 6 themes, 21 sub-themes, and 40 codes were extracted. The six themes included “benefit of work on health and life”, “adaptation to a new work environment”, “endurance”, “continuous efforts to maintain health”, “difficulties in self-management”, and “requirements for health promotion” (Table 2). 

### 3.1. Benefit of Work on Health and Life

#### 3.1.1. Vitality and Happiness 

Many participants reported that they found vitality in their lives by working, as working helped them alleviate boredom and provided a chance to interact with other people at work. They also expressed feeling happiness and a sense of accomplishment when they set and achieved goals.

“I find vitality by coming to work, and I have fun while cleaning up for someone and find happiness from it.” (Participant 11)

“Getting paid is great, but working provides vitality and helps me keep healthy, so I like it.” (Participant 1)

“I don’t know about others, but for me coming to work and talking, joking, and laughing with the other women at work is a source of happiness.” (Participant 4)

“It’s good to work. I feel like my health deteriorates if I just sit at home bored because I don’t have any reason to go out.” (Participant 7)

#### 3.1.2. Health Benefits 

Most participants maintained a regular daily schedule of going to bed early and waking up early to keep working. One of the aims of working was found to be maintaining health, as participants reported that working helped them maintain their health.

“In my opinion, if you’re healthy, you will stay healthier if you work. You might be unwell, but once you go to work, you forget about it. I would be sick, but when I go to work, I manage to find the strength to work. Then I forget about feeling sick. It happens like that.” (Participant 10)

#### 3.1.3. Gratitude and Pride

Participants felt thankful to be able to work, and felt pride that they worked when they compared themselves to people around them who do not work. Participants expressed that they did their best with the work given to them so they could continue working.

“I always feel so grateful when they extend my contract in January. I promise myself I will work hard for the year. Once the year goes by, I feel the same way in each January and work for another year. That has continued until now. So, I like working.” (Participant 1)

#### 3.1.4. Economic Independence 

Many participants reported that they worked for economic reasons, and that they worked to maintain a living. The reasons for working at an old age were so that they would not be a burden to their children and that they could continue to live independently during their senior years.

“Since I make my own money, I do not feel any shame. If not, if I don’t make money and my children give me money, it would be burdensome, and it would not be in line with what I was thinking when I first started working.” (Participant 1)

“Money is important. I’m not saying it is not important, but I put more meaning on being able to move and be active. If I say money is not important, I would be lying. To be honest.” (Participant 4)

### 3.2. Adaptation to a New Work Environment

#### 3.2.1. New Beginning 

Participants worried about their life after retirement as life expectancy increases. They opted to find new work after retirement. As they adapted to their new work, they felt like they were starting a new chapter of their lives.

“I did not work for a year after I retired. After not working for a year, I thought, if the life expectancy is climbing towards 100, and I am 60, I have another 30 years to live. To live 30 years, I need to earn money. I also need to think about my health. I can have some hobbies as I work in a new environment. It’s like I started a second chapter of my life.” (Participant 2)

#### 3.2.2. Adjusting to Shift Work 

Participants whose jobs involved shift work, such as security and healthcare workers, found shift work to be challenging at first, but they were able to adapt and no longer found shift work to be difficult. Elderly workers accepted and adapted to difficult work environments, and felt satisfaction in doing so.

“I am used to night shifts. It’s okay for me now. I was able to adjust, but young people these days do not want to take night shifts.” (Participant 12)

“It was difficult at first. But it’s been a while and it became a pattern, so it’s okay now.” (Participant 7)

### 3.3. Endurance

#### 3.3.1. Reason to Endure Suffering 

Participants experienced physical and psychological difficulties while working. The reason that they endured these difficult situations was to continue working in the future. Participants had complaints about their circumstances at work, but since it would be more difficult to find other work, they endured the difficulties.

“I need to do it even if it is hard. I can’t lose this job.” (Participant 12)

“I thought about doing something else. I thought about it, but what would I do? At this age, who will employ me? Just keep doing what I’m doing now, and if it comes to an end, it comes to an end. Where would I go, and what would I do? I can’t.” (Participant 9)

#### 3.3.2. Enduring Physical Symptoms

Physical pain was one of the difficulties that participants experienced while working, such as knee pain when participants had to sit at work, and sciatica when they had to lift something. Chronic pain was often endured or dealt with using pain killers.

“I hurt in a lot of different places. I can still manage, and if it gets worse, I go to the doctor’s office. We all endure some pain, right? We don’t always go to the doctor’s office. It’s like that.” (Participant 4)

#### 3.3.3. Persevering through Psychosocial Stress

Although social relationships at work can be pleasurable, some participants experienced stress from interpersonal relationships from time to time, resulting in psychological anxiety and insomnia. Interpersonal stress was seen as something participants could not control, so they simply endured it. One participant was taking medication for anxiety and insomnia, but feared developing a tolerance to the drug if taken regularly; therefore, the participant endured the symptoms.

“Relationships with other people are the most stressful. People are all different, so I just need to be patient and endure.” (Participant 11)

“I slept well when I took half a pill. But once I continued the drug became less effective. […] Even if I sleep for a little bit, I am depending on the medicine. So even if I sleep very lightly, I prefer not to depend on the drug so much. So, I haven’t been sleeping well. It’s been a while since I have.” (Participant 4)

### 3.4. Continuous Efforts to Maintain Health

#### 3.4.1. Motivations for Self-Management 

Elderly workers continuously made efforts to maintain their health. They maintained their health so that they could keep working, recognized health as their top priority, and tried not to depend on family members. Participants who experienced a family member’s deteriorating health or death or their own health problems expressed a stronger will to manage their health as they understood the value of health. Some participants also recognized that their health had worsened over time, and thus made more efforts to maintain their health.

“Health is life, and life is health. Health is the best. So, best not to be ill. It’s pathetic to lay in bed and be sick when you’re old. So, I think to myself, I need to manage my health.” (Participant 11)

“I should put efforts into maintaining my health and living a healthy life. When I look at my parents, I think to myself, I should not be a burden to my family.” (Participant 5)

“My children inherit my illness. If children have to take care of me, it’s difficult for all of us. If I’m healthy, I’m helping my children.” (Participant 8)

#### 3.4.2. Physical Self-Management

Most participants reported that they visited doctor’s offices regularly or got a check-up every year or two in order to manage their physical health. Participants diligently followed guidance from doctors regarding medication, diet, and exercise, and were taking several supplements including omega-3, lutein, vitamins, minerals, and probiotics. Participants sought alternative treatments that they found effective for pain alleviation based on their experiences (e.g., acupuncture and herbal medicine). To maintain health, they ate a plant-based diet and regularly engaged in walking, aerobics, and muscle-strengthening exercises. They also adopted healthy lifestyles by not drinking, not smoking, and keeping a regular schedule.

“I need to take medicine every day. I also need to go to the doctor’s office on the appointment day. At the doctor’s office, my blood pressure is measured, and if it is normal, I get 2 months’ worth of medicine. If it is high, I get medicine for 1 month and then go back to check again. At the hospital. I get checked from time to time.” (Participant 4)

“I don’t eat salty food because they say salty food makes you drink a lot of water and increase your blood pressure as a result. Maybe because I don’t eat salty food, my blood pressure is okay these days.” (Participant 8)

“I exercise a lot. First, muscle-strengthening exercises. I do a lot of muscle-strengthening exercises, and I also walk a lot.” (Participant 2)

“Keeping a regular schedule. I take blood pressure medicine in the morning, take omega-3 and other supplements after lunch, and again after dinner. I do this regularly. I never skip and am very precise.” (Participant 11)

#### 3.4.3. Psychological Self-Management

To manage their psychological health, participants tried to think positively and drew boundaries with coworkers to relieve work stress. Participants felt supported by maintaining a close relationship with a spouse or a friend, traveled to brighten their mood, and relied on religion.

“When that happens, I cope by being happy. There’s no other choice. Only happiness. If I start complaining, I can’t do it. I can’t work if I constantly complain. If someone suggests I do something, I comply and have fun.” (Participant 9)

“I talk with my husband and get advice from him. After coming back from work every day, I look back on my day and think whether I did wrong by anyone I took care of. I reflect on it.” (Participant 10)

“(When I get stressed) I pray and try to relax.” (Participant 5)

#### 3.4.4. Efforts to Obtain Health Information 

When participants saw their friends or other people around them manage their health, participants felt inspired to manage their health. Gaining information through friends or TV and the internet was found to be helpful to self-management.

“Self-management is not really difficult. In order for self-management to be a habit and lifestyle, one must be prepared.” (Participant 8)

“If I don’t do it myself, there’s no other way. I need to do it on my own.” (Participant 10)

“I have a friend who is younger than me, but that friend is forgetful and started taking medicine for prevention dementia. So I started taking the medicine as well.” (Participant 3)

### 3.5. Difficulties in Self-Management

#### 3.5.1. Time 

When discussing barriers to self-management, the fact that participants did not have enough time and that they could not participate in any health promotion program in a community setting, as those programs were usually during work hours, was mentioned most often. Participants felt regret about not being able to participate in these health programs.

“The timing also doesn’t work out. Most welfare centers operate in the morning and close by 7 or 8 p.m. Working people like me cannot go even if we want to. […] When I’m done at work and return home, it’s dinner time. Most people eat dinner and go to bed by 7–9 p.m. Most people I ask say they don’t have enough time to exercise. Some have to wake up at 2 am or 4 a.m. So, a lot of people go to bed by 8 p.m. or 9 p.m. They need to go to work at 5 am. So they can’t manage their health.” (Participant 2)

“The timing isn’t right for me. I’m done here at 3:30 p.m., and when I go there, people are already done. It’s around 4:30 p.m. when I get there. Most people leave by 4 p.m. or 5 p.m. I can’t do it because of the timing.” (Participant 4)

#### 3.5.2. Poor Work Environment

Elderly workers experienced their health deteriorating because of poor conditions in their workplace. Their level of fatigue increased because they did not have adequate space to rest after physical labor.

“We have to eat food in tiny spaces. Rests are very short. The space is not big enough.” (Participant 3)

“(My workplace is) on the second floor of the basement, and it’s just like a storage space, so the air quality is bad. […] The resting area is very bad. When I enter, I feel my voice change instantly. I also cough. I’d be fine in the morning and start coughing as soon as I go in to work. You know, like foggy pollution.” (Participant 4)

#### 3.5.3. Physical Fatigue after Work

Participants did not exercise since they wanted to rest after doing physical labor. They also felt too tired to prepare for meals, so they ate simply.

“I can’t be bothered to. Because I work like this during the day, I don’t exercise because I can’t be bothered to.” (Participant 3)

“I can squeeze out time to exercise, but it’s not easy to feel up for it. After work, I feel tired, so I just feel, I don’t want to go today.” (Participant 11)

#### 3.5.4. Social Environment

Participants expressed that they would like to do some simple exercises such as walking or stretching during work, but they felt self-conscious. Other barriers included living alone and decreased social interactions, as it was hard to find someone to exercise with due to limited social contacts.

“Honestly, I feel self-conscious, and I don’t know what other people will think when they see me.” (Participant 1)

“Because I don’t have anyone around me, I find it difficult to go to places to exercise.” (Participant 12)

#### 3.5.5. Cost and Distance 

Other barriers identified were fees required to exercise and accessibility in terms of distance to the closest exercise facility.

“To do anything, you need money. So, I end up not being able to exercise. […] I have the intention to go, but if it’s far, I can’t go.” (Participant 12)

### 3.6. Requirements for Health Promotion

#### 3.6.1. Need for Exercise Equipment and Space

Participants voiced that they needed exercise equipment that they could use conveniently and exercise space for use after work.

“I wish there would be a place senior citizens can go to exercise at any time. A space where we can exercise and meet other people and chat. That would be great.” (Participant 1)

“At least running machines, walking equipment. I would like one of those options.” (Participant 11)

#### 3.6.2. Need for Educational Programs for Self-Management 

The education that participants receive at the workplace is limited to required safety education, and there are no health programs for employees. Participants wanted education about chronic diseases and recreational activities such as singing classes to relieve workplace stress.

“If there are places we can go to, such as singing classes…if we can use these services more frequently, we can go for even 30 min or an hour, sing along, and have a fun time. I think that would be good.” (Participant 3)

“When I sometimes look at what’s happening on the first floor, there are programs such as ‘About diabetes.’ I really want to go.” (Participant 1)

#### 3.6.3. Identifying Time for Self-Management through Flexible Work Hours

Participants regretted not being able to participate in any health promotion program in a community setting. These programs are usually scheduled during the working time (9:00 to 18:00), and elderly workers therefore experienced difficulties attending them. Thus, participants voiced the need for flexible time management or health programs that take place after work hours.

“If our hours are shorter, there will be some time for self-management. Time for exercise. Even just for an hour, then I will exercise a bit more.” (Participant 3)

“It seems like the programs are only for elderly who stay at home. So, if there are more various times, for example 6 p.m. or 7 p.m., that would be great.” (Participant 1)

## 4. Discussion

The aim of this study was to explore the meaning of work and health-related self-management experiences of elderly workers with multiple chronic diseases. Elderly workers proactively managed their chronic diseases, reaffirmed their will to live through work, and adapted to new work environments. It was shown that their lives were independent and imbued with positive attitudes as they embarked upon new chapters of their lives, despite suffering from their chronic diseases and the aging process.

Participants of this research continued to work for economic reasons, but most retired from their previous workplace and found new blue-collar jobs. Consistent with previous studies reporting that most elderly in South Korea re-started work in blue-collar positions [6], most of the study participants were working as sanitation or security workers. Paradoxically, elderly workers felt the benefits of work such as vitality, health, and social relationship, but simultaneously expressed difficulties derived from work such as physical difficulties due to aging and emotional difficulties at the workplace. Nevertheless, elderly workers adjusted to lifestyle changes due to shift work and endured poor work environments to maintain a living and to avoid becoming a burden to their family. In traditional Korean cultures, children tended to be responsible for supporting their parents [17]. This aspect of culture has emerged as a social issue along with increases in life expectancy. According to a study by the Korea Institute for Health and Social Affairs, children support their parents when their parents are ill (27%) and when their parents are not financially capable (21.9%) [17]. Since supporting parents is a pervasive pattern in Korean society [17], the elderly workers who participated in this study did not want to burden their children with the obligation to provide financial support to their parents. This may be the reason why the older adults in this study engaged in economic activities to earn their living expenses [6]. The participants in this study viewed their lives positively despite difficult circumstances and had a strong will to adjust to their changing environments. They reported that the benefits of working outweighed the difficulties, and that work allowed them to be independent. 

It is known that aging workers experience adjustment issues at work [18]. However, the elderly have higher emotional wellness, as their adaptive strategies are more enhanced than those of younger individuals [19]. In a study of laborers, age was found to be associated with higher levels of positive emotions [20]. The participants of this study made efforts to adjust to their new work environment, felt proud of being able to work at their age, and gained energy through work. Moreover, in order to manage stress from work, they engaged in positive thinking, gained social support from spouses or friends, and adopted other strategies such as traveling and engaging in religious activities. They demonstrated their experience by relieving stress using methods that they had learned through previous experiences and continuously put in hard work to adjust to their workplace. Elderly workers perceived work as an important aspect of their lives, and referred to work as their life. Their positive attitudes helped them adapt to the work environment.

For elderly workers, it is a challenge to adjust to the physical and cognitive changes that accompany aging. Many can become overwhelmed, and their health outcomes can deteriorate [18]. Previous studies found that elderly workers with health problems were anxious about having to stop working [21], but the participants of this study continued working while proactively managing their health since the severity of their diseases was low, even though they had an average of 2.83 chronic diseases. However, lack of time, a poor work environment, including a lack of rest areas, physical fatigue after work, weak social connections, fees, and distance were identified as barriers to health-related self-management. As the workforce ages, and considering that more than half of elderly workers have multiple chronic diseases [6], it is possible that aspects of the work environment may cause the health status of elderly workers to deteriorate and interfere with their self-management of chronic diseases [11]. Since the number of elderly individuals participating in the labor market will increase concomitantly with the aging population and increasing life expectancy, programs that consider the health-related characteristics of elderly workers and their work environment are necessary.

The participants in this study acknowledged the benefit of economic independence through work, but expressed discomfort caused by multiple chronic diseases and aging at the same time. Most elderly workers who work in low-wage blue-collar jobs in South Korea have annual employment contracts [6]. Thus, elderly workers were afraid that they would lose their jobs because of poor health [18]. However, they did not actively visit doctor’s offices or take time to take care of their health, and endured physical and psychological discomfort. They may have perceived frequent visits to doctor’s offices as an indicator that their health condition was worsening. This phenomenon of endurance is also shaped by socio-cultural factors. Pain experiences and psychological processes associated with pain may differ across ethnic groups [22]. People from Eastern cultures have been reported to have higher pain tolerance than those from Western cultures [23], and “chronic” diseases can be perceived as difficult to cure even if treated. Thus, healthcare providers consider elderly workers’ vulnerabilities in terms of their physical condition and work environment [10], and should encourage active participation in health care. 

Additionally, how one deals with pain depends on one’s level of education [24]. The level of education of the participants in the present study varied, but those who expressed that they endured chronic pain or psychological stress tended to have relatively low education levels. Therefore, the endurance of physical and psychological suffering needs further research in consideration of socio-cultural factors and educational level.

The main strength of this study is that the meaning of work and health was investigated among elderly workers who were living new chapters of their lives through in-depth interviews about their experiences of self-management. The difference of this study compared to existing studies is that this study revealed the positive energy of elderly workers, who adjusted physically, mentally, and socially despite poor working conditions. 

This study had several limitations. First, this study was conducted in two large cities (Seoul and Daejeon) in South Korea. Depending on the socio-cultural environment, the meaning of work or health-related self-management experiences may differ, so it is recommended to conduct repeated studies in various regions and cultures in the future. Second, it is suggested that researchers use a mixed-methods design to analyze factors affecting health-related self-management or the health care needs of elderly workers through a quantitative method in a future study. 

## 5. Conclusions

The results of this qualitative study indicate that elderly workers gained vitality through work, but were stressed about the difficulties that accompany aging. Elderly workers tried hard to overcome these difficulties. In the future, research on health-related self-management experiences among elderly workers should be conducted in various sociocultural settings.

## Figures and Tables

**Table 1:** General characteristics of participants.

ID	Gender	Age	Residence	Education	Occupation	Subjective Socioeconomic Status	Religion	Household Members	Number of Chronic Diseases
1	Female	72	Daejeon	High school	Sanitation worker	Middle	Catholicism	None	2
2	Male	65	Daejeon	University	Security guard	Middle	Christianity	Spouse	3
3	Female	72	Daejeon	Elementary school	Sanitation worker	Low	Christianity	Unmarried child	3
4	Female	67	Daejeon	Elementary school	Sanitation worker	Low	Buddhism	Spouse	3
5	Male	67	Seoul	High school	Office worker	Middle	Christianity	Spouse	2
6	Male	65	Seoul	Middle school	Business owner	Middle	Christianity	Unmarried child	3
7	Male	74	Seoul	Middle school	Security guard	Middle	Christianity	Unmarried child	2
8	Male	73	Seoul	High school	Security guard	Middle	Christianity	Unmarried child	2
9	Female	68	Seoul	Elementary school	Sanitation worker	Middle	Christianity	Spouse	4
10	Female	65	Seoul	Elementary school	Certified nursing assistant	Middle	Catholicism	Married children	3
11	Female	68	Daejeon	Elementary school	Sanitation worker	Low	Christianity	None	4
12	Female	65	Daejeon	Elementary school	Sanitation worker	Low	Buddhism	None	3

**Table 2:** Outcome of qualitative analysis.

Theme	Sub-Theme
Benefit of work on health and life	● Vitality and happiness ● Health benefits ● Gratitude and pride ● Economic independence
Adaptation to a new work environment	● New beginning ● Adjusting to shift work
Endurance	● Reason to endure suffering ● Enduring physical symptoms ● Persevering through psychosocial stress
Continuous efforts to maintain health	● Motivations for self-management ● Physical self-management ● Psychological self-management ● Efforts to obtain health information
Difficulties in self-management	● Time ● Poor work environment ● Physical fatigue after work ● Social Environment ● Cost and distance
Requirements for health promotion	● Need for exercise equipment and space ● Need for educational programs for self-management ● Identifying time for self-management through flexible work hours

## Appendix A

**Table A1:** Interview guide.

Main Questions
What does work mean to elderly workers?
What does health mean to elderly workers?
What are the experiences of elderly workers with multiple chronic diseases?
What are the facilitators of self-management of chronic diseases among elderly workers?
What are the barriers to self-management of chronic diseases among elderly workers?
What are the requirements for self-management among elderly workers?
